# Supplementary material for: End Sequence Analysis Toolkit (ESAT) expands the extractable information from single-cell RNA-seq data
Source: Genome Res. 2016 Oct;26(10):1397–410. doi: 10.1101/gr.207902.116 (PMC5052061; doi:10.1101/gr.207902.116)
Supplement: Supplemental Material [file supp_gr.207902.116_Supp_Materials.tar.gz › ESAT-esat_v0.1/doc/html/classbroad_1_1core_1_1multiplealignment_1_1_alignment_extractor.html]

ESAT: broad.core.multiplealignment.AlignmentExtractor Class Reference


ESAT


- Main Page
- Related Pages
- Packages
- Classes
- Files

- Class List
- Class Index
- Class Hierarchy
- Class Members

All Classes Namespaces Files Functions Variables Enumerator Pages

- broad
- core
- multiplealignment
- AlignmentExtractor

Classes |
Static Public Member Functions |
Static Public Attributes |
List of all members

broad.core.multiplealignment.AlignmentExtractor Class Reference

Collaboration diagram for broad.core.multiplealignment.AlignmentExtractor:

[legend]

|  |  |
| --- | --- |
| Static Public Member Functions | |
| static void | main (String[] args) throws IOException, IllegalAccessException, ParseException |
|  | |
| static void | extractGFFRegions (String alignmentFile, String outputFile, String gff, String inFormat, String outFormat, String[] seqs) throws IOException, ParseException |
|  | |

|  |  |
| --- | --- |
| Static Public Attributes | |
| static ArrayList< String > | degenerateCodonStarts |
|  | |
| static ArrayList< String > | twoFoldDegenerateCodons |
|  | |
| static ArrayList< String > | threeFoldDegenerateCodons |
|  | |
| static ArrayList< String > | nonDegenerateCodons |
|  | |
| static HashMap< String, String > | codonToAminoacid |
|  | |
| static String | USAGE |
|  | |

## Member Function Documentation

|  |  |  |  |
| --- | --- | --- | --- |
| static void broad.core.multiplealignment.AlignmentExtractor.extractGFFRegions | ( | String | *alignmentFile*, |
|  |  | String | *outputFile*, |
|  |  | String | *gff*, |
|  |  | String | *inFormat*, |
|  |  | String | *outFormat*, |
|  |  | String[] | *seqs* |
|  | ) |  | throws IOException, ParseException |

static

Here is the call graph for this function:

Here is the caller graph for this function:

|  |  |  |  |  |  |
| --- | --- | --- | --- | --- | --- |
| static void broad.core.multiplealignment.AlignmentExtractor.main | ( | String[] | *args* | ) | throws IOException, IllegalAccessException, ParseException |

static

Here is the call graph for this function:

## Member Data Documentation

HashMap<String, String> broad.core.multiplealignment.AlignmentExtractor.codonToAminoacid

static

ArrayList<String> broad.core.multiplealignment.AlignmentExtractor.degenerateCodonStarts

static

ArrayList<String> broad.core.multiplealignment.AlignmentExtractor.nonDegenerateCodons

static

ArrayList<String> broad.core.multiplealignment.AlignmentExtractor.threeFoldDegenerateCodons

static

ArrayList<String> broad.core.multiplealignment.AlignmentExtractor.twoFoldDegenerateCodons

static

String broad.core.multiplealignment.AlignmentExtractor.USAGE

static

**Initial value:**

= "Usage: AlignmentExtractor TASK=<task\_num> <task\_args>\n" +

"\tTasks:\n" +

"\t\t1. Extract Regions: IN=<Multiple alignment file> OUT=<output file> ANNOTGFF=<GFF annotation file, sequence name should match one of the aligned sequence IDs> INFORMAT=<[FASTA]/EXON/PHYLIP/SEQPHYLIP> OUTFORMAT<[FASTA]/EXON/PHYLIP> SEQS=<comma separated list of sequence ids>\n" +

"\t\t2. Extract 4D sites from exon alignment: IN=<Exon multiple alignment> OUT=<output file> INFORMAT=<input format (default FASTA)> OUTFORMAT=<output format (default FASTA)>\n " +

"\t\t3. Extract Conserved sites from exon alignment: IN=<Exon multiple alignment> OUT=<output alignment file> INFORMAT=<input format (default FASTA)> OUTFORMAT=<output format (default FASTA)>\n"+

"\t\t4. Extract region IN=<Multiple alignment file> OUT=<output file> REGION=<start..end> REF=<reference> INFORMAT=<[FASTA]/EXON/PHYLIP> OUTFORMAT<[FASTA]/EXON/PHYLIP/SEQPHYLIP> \n" +

"\t\t5. Convert alignment file IN=<Multiple Alignment File> OUT=<output file> INFORMAT=<[FASTA]/EXON/PHYLIP> OUTFORMAT<FASTA/EXON/[PHYLIP]/SEQPHYLIP> [-compress <if the output alignment should be reference gap free>] \n" +

"\t\t6. Introduce Random Gaps -in <Multiple Alignment File default is standard input> -out <output gapped alignment defalut is standard out> -informat <[FASTA]/EXON/PHYLIP> -outformat <FASTA/EXON/[PHYLIP]/SEQPHYLIP> -maxGaps <maximum number of gaps> [-ref <reference if no gaps should be introduced in reference>]\n" +

"\t\t7. Permute Columns -in <Multiple Alignment File default is standard input> -out <output gapped alignment defalut is standard out> -informat <[FASTA]/EXON/PHYLIP> -outformat <FASTA/EXON/[PHYLIP]/SEQPHYLIP> " +

"\n\t8. Sample Columns from Alignment -in <Multiple Alignment File default is standard input> -out <output gapped alignment defalut is standard out> -informat <[FASTA]/EXON/PHYLIP> -outformat <[FASTA]/EXON/PHYLIP/SEQPHYLIP> -cols <Number of columns to sample> [-consecutiveCols <Sample consecutive columns rather than single ones>]" +

"\n\t9. Combined alignments -informat <[FASTA], PHYLIP, SEQPHYLIP> -outformat <[FASTA], PHYLIP, SEQPHYLIP> -in <Standard input of file with a list of alignment files> -out <standard output or file name> [-maxColumns <If specified, and the combined alignment is larger than this value, a sampled alignmnet will be generated instead of the combined one>]" +

"\n"

---

The documentation for this class was generated from the following file:

- java/broad/core/multiplealignment/AlignmentExtractor.java


---

Generated on Fri Aug 15 2014 15:10:54 for ESAT by  

 1.8.7
